# State-of-the-Art on Brain-Computer Interface Technology

**DOI:** 10.3390/s23136001

**Published:** 2023-06-28

**Authors:** Janis Peksa, Dmytro Mamchur

**Affiliations:** 1 Department of Information Technologies, Turiba University, Graudu Street 68, LV-1058 Riga, Latvia; dgmamchur@gmail.com; 2 Institute of Information Technology, Riga Technical University, Kalku Street 1, LV-1658 Riga, Latvia; 3 Computer Engineering and Electronics Department, Kremenchuk Mykhailo Ostrohradskyi National University, Pershotravneva 20, 39600 Kremenchuk, Ukraine

**Keywords:** brain–computer interface, EEG, artificial intelligence, classification, signal processing

## Abstract

This paper provides a comprehensive overview of the state-of-the-art in brain–computer interfaces (BCI). It begins by providing an introduction to BCIs, describing their main operation principles and most widely used platforms. The paper then examines the various components of a BCI system, such as hardware, software, and signal processing algorithms. Finally, it looks at current trends in research related to BCI use for medical, educational, and other purposes, as well as potential future applications of this technology. The paper concludes by highlighting some key challenges that still need to be addressed before widespread adoption can occur. By presenting an up-to-date assessment of the state-of-the-art in BCI technology, this paper will provide valuable insight into where this field is heading in terms of progress and innovation.

## 1. Introduction

Brain–computer interfaces (BCIs) are a rapidly evolving technology that has the potential to revolutionize how humans interact with computers [1,2,3,4]. BCIs measure brain activity and translate it into commands for a computer or other device, allowing users to control machines and devices using only their thoughts. Neurogadgets, ranging from moving robotic spiders and balls to more practical applications, are increasingly being used for entertainment purposes. However, what is more important is that neurogadgets are also being developed to assist people with disabilities, such as those with paralysis of the limbs [5,6,7,8,9,10].

BCIs are typically divided into unidirectional and bidirectional categories based on the direction of their action. Unidirectional BCIs either receive signals from the brain or send them to it, while bidirectional BCIs allow for information exchange in both directions, enabling control of external devices by the brain.

Research into feedback methods is ongoing, with the aim of developing technologies that can transform external commands into electrical signals transmitted via the nervous system. For instance, it could be used to enable electrical stimulation of leg muscles in people with spinal cord injuries, allowing them to regain mobility by controlling their movements through a tablet device [11].

The utilization of neural networks and other learning algorithms in signal processing is commonplace, as brain activity varies between individuals. Consequently, these systems require lengthy training sessions to enable the BCI to accurately interpret commands from a particular user. The duration of the training depends on the number of commands received by BCI.

While this technology is still in its early stages of development, recent advances have shown great promise for applications ranging from medical rehabilitation to gaming and entertainment. This paper will provide an overview of the current state-of-the-art in BCI technologies, discussing various platforms, techniques, and applications currently being explored.

## 2. Platforms

The operation of the interface is typically structured in this manner: electrodes detect brain signals, which are then processed by an BCI microcontroller to remove any noise or artifacts caused by both external and device-specific factors. Subsequently, the obtained signal is analyzed to identify the corresponding command; artificial neural networks are often utilized for this purpose due to their high data processing and adaptation capabilities.

**Figure 1:**
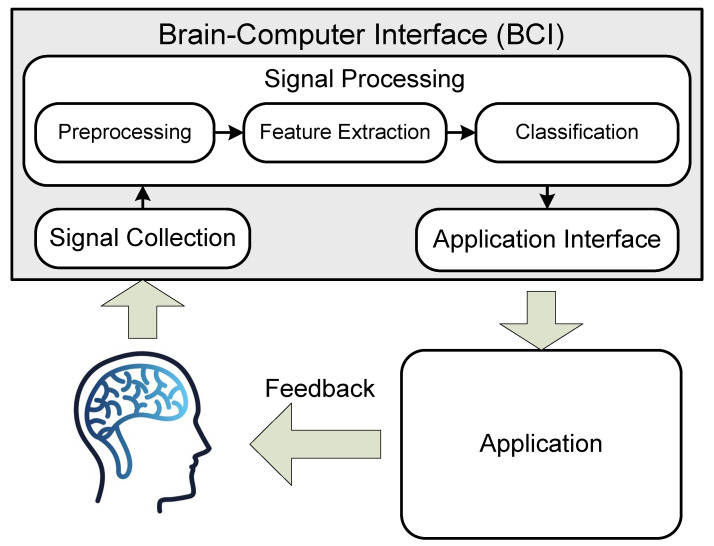
BCI operation principle.

The detected command is usually sent to an external device for further processing according to a pre-programmed algorithm, although highly specialized systems may assume this task themselves. Ultimately, the received command is interpreted as per its specific characteristics on the controlled device (Figure 1). The principles underlying BCI operation are described in [12,13,14,15].

**Figure 2:**
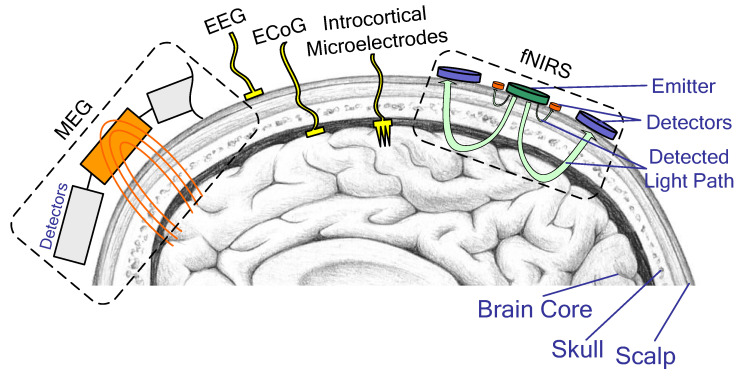
BCI sensor mounting types: invasive (IM), semi-invasive (ECoG), and non-invasive (MEG, EEG, fNIRS).

According to the degree of invasiveness, neural interfaces are divided into three categories: invasive, non-invasive, and semi-invasive. Invasive neural interfaces require direct implantation of intracortical microelectrodes (IM) into the human brain, providing the highest efficacy but posing a greater risk. Non-invasive neural interfaces analyze brain activity from the surface of the head by using electroencephalography (EEG), magnetoencephalography (MEG), or functional magnetic resonance imaging without implanting electrodes. Semi-invasive BCIs have electrodes located under the skull bone on the surface of the brain, such as electrocorticography (ECoG) (Figure 2).

At present, invasive and semi-invasive neurointerfaces are mainly utilized in medical contexts to enhance the wellbeing of individuals with disabilities. Additionally, these devices are also being used to correct and prevent a variety of diseases. On the other hand, non-invasive neural interfaces have been gaining traction in the gaming industry [16,17,18,19]. As more types of neurogadgets become available, it is possible that this sector will experience a revolution. It is envisaged that smartphones may be able to record human thoughts in the foreseeable future; research into this area has been ongoing [20,21].

The most common platform used for BCI research is electroencephalography [22,23,24,25,26]. EEG measures electrical signals produced by neurons within the brain through electrodes placed on the scalp, providing researchers with detailed information about neural activity associated with different cognitive functions. Other platforms commonly used include functional near-infrared spectroscopy (fNIRS) [27,28,29,30,31,32,33], magnetoencephalography [34,35,36], and electrocorticography [37,38,39]. These methods measure different types of neural signals than EEG but can still be useful in developing effective BCI systems due to their higher temporal resolution or ability to detect deeper sources of brain activity.

All these platforms have their own pros and cons, which are analyzed below.

### 2.1. EEG Platform

The electroencephalogram is a widely used tool for monitoring electrical activity in the brain. EEG signals, which are a visual representation of the frequency activity of the human brain [40,41], are commonly used as inputs for BCI systems. It has been used to diagnose and treat neurological diseases, monitor sleep patterns, and study cognitive processes such as attention and memory. In recent years, advances in technology have enabled the development of EEG sensors that are smaller, more accurate, and easier to use than ever before. Due to its non-invasive data collection principle and relatively simple signal interpretation, this platform is one of the most commonly used BCI techniques nowadays.

EEG sensors measure electrical activity produced by neurons in the brain using electrodes placed on the scalp or other parts of the body. By monitoring this activity over time, clinicians can detect changes associated with different mental states, such as sleep or alertness. Additionally, certain types of abnormal brain activity can be detected through EEG readings; these may include seizures or evidence of stroke-related damage. The data collected from EEG recordings can also be analyzed to assess cognitive abilities such as attention span or memory recall speed.

**Figure 3:**
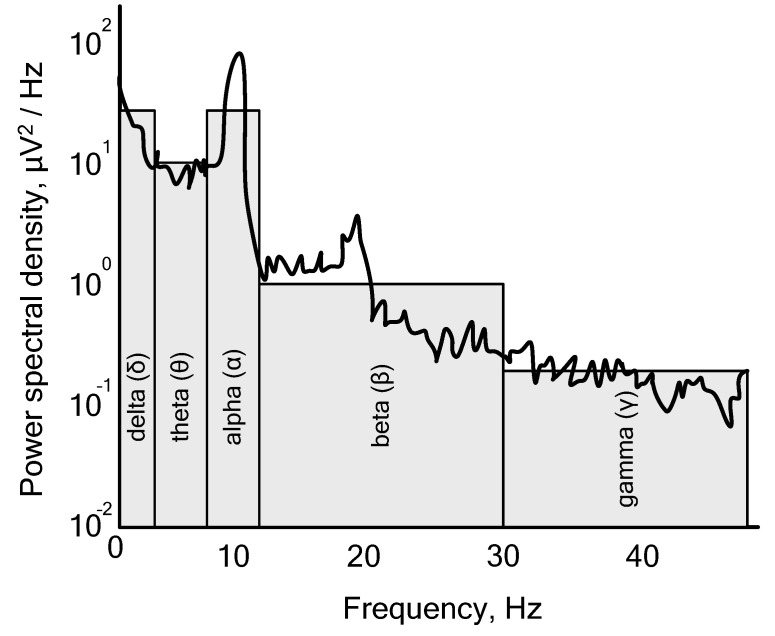
An example of the frequency spectrum of a human brain electroencephalogram.

Figure 3 illustrates that there are four distinct “rhythms” of the human brain, which can be categorized based on their frequency: δ delta (0.1–4 Hz), θ theta (4–7.5 Hz), α alpha (7.5–12 Hz), β beta (12–30 Hz), and γ gamma (over 30 Hz). It is important to note that these rhythms differ in amplitude as well as frequency.

Recent advances in EEG sensor technology have made them smaller, lighter, and cheaper than ever before while still providing high levels of accuracy and reliability when compared to traditional systems [25,26,42]. Moreover, newer systems often make use of dry electrode designs, which eliminate the need for conductive gels that were previously required for proper functioning [43,44,45,46,47]. Furthermore, many new portable devices exist now that allow users to easily record their own EEG signals without having to visit a clinic [9,48,49,50,51]. These devices typically employ Bluetooth connectivity so that they can transmit their data wirelessly directly to computers for analysis. Recent advancements have also led to improvements in signal processing algorithms, which enable better detection and analysis techniques [52,53,54]. For example, it is now possible to detect subtle changes within short periods of time, leading to improved diagnosis capabilities. Additionally, some algorithms are able to identify distinct features within each individual’s recordings, allowing personalized treatment approaches [55,56]. Finally, artificial intelligence techniques are being explored that could help automate certain aspects of processing raw data, resulting in faster diagnostic times [57,58,59,60].

The overall quality of an EEG signal is affected by both the quantity and placement of electrodes. Increasing the electrode count can improve spatial resolution, allowing for more detailed analysis. Additionally, a greater number of electrodes allows for better noise reduction techniques such as averaging or interpolation. However, increasing electrode count also increases the cost and complexity associated with recording equipment; thus, it is important to consider tradeoffs between benefits in accuracy versus the burden imposed by additional hardware requirements when choosing an optimal sensor configuration.

In addition to overall quantity, positioning plays a critical role in determining signal quality during EEG recordings. Different positions provide varying levels of information about different parts of the brain; therefore, careful consideration must be taken when selecting which sites should be used for data acquisition. Furthermore, differences in skull thickness across individuals may require adjustments from standard placements due to potential changes in impedance at various locations relative to one another. It is also important that all channels are placed symmetrically with respect to each other so that any artifacts generated from movement will cancel out. Finally, proper reference placement is necessary since it serves as a “ground” against which all other signals can be compared. The effectiveness of an EEG system relies heavily on the selection, number, and arrangement/placement of the sensors being used. Careful consideration must be given to these factors so that optimal results can be achieved while minimizing costs associated with hardware requirements. With advances in technology continuing apace, it will become increasingly possible to optimize sensor configurations, further improving BCI performance capabilities over time.

### 2.2. Other Platforms

Functional near-infrared spectroscopy is a non-invasive brain imaging technique that uses light to measure changes in the concentration of oxygenated and deoxygenated hemoglobin in the brain [28,30,31,33,61]. fNIRS can be used to measure both regional and global activity in the cortex, allowing for real-time monitoring of neural activity. The technology has been applied to BCI applications such as motor imagery, language processing, affective state recognition, and EEG/ERP source localization. As with EEG, the number and arrangement of sensors are important considerations. If too few sensors are used, then not enough information may be collected to accurately assess brain activity; conversely, if too many sensors are employed, they may interfere with each other or saturate certain areas due to excessive light intensity. The placement and orientation of each sensor must be carefully considered so that it is able to detect meaningful signals from its target area without being overly influenced by nearby sources. Placing one sensor too close to another could lead to interference between them or cause one sensor’s signal strength to dominate over another’s. Furthermore, depending on the type of task being performed during an experiment, there may be different requirements regarding how many channels should be monitored at once as well as where those channels should be located relative to each other. Therefore, careful consideration must go into deciding how many channels should be included in any given experiment and where they should be best placed in order to ensure accurate data collection while avoiding unnecessary noise or saturation effects.

Magnetoencephalography is a non-invasive brain imaging technique that measures magnetic fields generated by electrical currents inside neurons [12,35,36,62,63]. It provides high temporal resolution with excellent spatial accuracy and can be used to track changes in neural activity related to cognitive processes such as attention or memory formation over time. MEG has been used for auditory BCI research but also shows potential for visual BCIs as well as multimodal approaches combining MEG with other modalities such as EEG or fMRI. One of the important factors affecting signal resolution is sensor density: more sensors mean more accurate localization of neural activity within the brain, allowing better control over output devices such as robotic limbs or computer cursors. Increasing sensor density above 30–50 sensors per cm^3^ can significantly improve spatial resolution and accuracy when compared to lower densities. However, this must be balanced against increased costs associated with higher numbers of sensors as well as any potential interference between closely spaced elements due to mutual inductance. In addition to sensor density, placement also plays an important role in determining signal fidelity. Ideally, each individual’s head should be modeled before placing electrodes so that they are optimally positioned based on their unique anatomy. Furthermore, optimal placement may vary depending on what type of information one wants to extract from recorded data. For example, if one wishes to study motor cortex activation, it would make sense to place electrodes near primary motor areas, while studying visual cortex activation might require different locations. Using multiple layers of overlapping grids can help reduce noise levels caused by external sources such as power lines or electronic equipment, although further research into this area is still needed before definitive conclusions can be drawn about its efficacy.

Electrocorticography is an invasive brain imaging technique that records electrical signals from the surface of the cerebral cortex directly through implanted electrodes placed on top of the cortical surface [38,39,64,65]. ECoG offers high temporal resolution (~milliseconds), excellent signal quality, very low noise levels, and direct access to underlying neuronal sources, which makes it particularly suitable for decoding complex mental states such as speech production or intention decoding from motor areas. However, due to its invasive nature, this method requires surgery, which limits its widespread use outside clinical settings. As well as with previously described platforms, increasing the total number of ECoG electrodes generally improves the overall accuracy rate across all task types, regardless of whether they are placed at random locations or at regular intervals around the cortex. However, accuracy rates do not improve significantly when more than eight regularly distributed electrodes are used. Furthermore, even though traditional EEG systems have higher overall accuracy rates than any individual ECoG setup due to their greater electrode coverage area, they are still outperformed by some smaller-scale ECoG setups under certain conditions—particularly when dealing with multi-task classifications involving complex patterns such as visual stimuli recognition.

### 2.3. BCI Platforms Comparison

Based on the provided analysis, we can draw the following conclusions.

EEG is a non-invasive technique that measures electrical activity in the brain via electrodes placed on the scalp. It has good temporal resolution, allowing it to detect changes in neural activity within milliseconds. EEG is relatively inexpensive and portable, making it well suited for use in BCI systems. EEG’s key issues are the following:

- Signal quality: EEG signals are highly sensitive to noise and artifacts, so it is important to ensure that the signal quality is optimal for BCI applications;
- Feature extraction: the ability to accurately extract meaningful information from raw EEG data is a key issue in BCI research as this determines how effective the system will be at recognizing user intentions and commands;
- Classification accuracy: designing efficient algorithms for classifying EEG signals into different categories (e.g., left vs. right hand movement) is an important issue in BCI research as it determines how well the system can recognize user commands or intentions.
- User interface design: designing user interfaces that are intuitive and easy to use is an important issue in EEG-based BCIs as it can determine how easily users can interact with the system;
- Adaptability: developing algorithms that can adapt to individual users’ brain activity and recognize subtle changes in EEG patterns is an important research topic for creating robust BCI systems;
- System reliability: ensuring reliable performance of a BCI system over long periods of time with minimal calibration or setup requirements is an important challenge in EEG-based BCIs due to the dynamic nature of brain activity and its variability across users and sessions.

fNIRS uses light to measure changes in oxygenated hemoglobin levels associated with neural activity. fNIRS offers excellent spatial resolution and can be used to monitor multiple areas of the brain simultaneously, but its temporal resolution is limited compared to other techniques such as EEG. The key issues of the fNIRS platform are the following:

- Signal quality: fNIRS signals are relatively weak and affected by noise, making it difficult to accurately detect changes in brain activity;
- Spatial resolution: the spatial resolution of fNIRS is limited due to the limited number of sources and detectors, which may lead to incorrect interpretations of the data;
- Temporal resolution: fNIRS has a relatively slow response time compared with other BCI modalities such as EEG or MEG, meaning that more complex cognitive tasks may not be suitable for this technology;
- Cost: while fNIRS systems are becoming increasingly affordable, they remain significantly more expensive than EEG or MEG systems and require specialized training in their use and interpretation of results;
- Safety: fNIRS systems operate by sending light into the head, which could potentially lead to eye damage if not used correctly.

MEG is an imaging technique that records magnetic fields produced by electrical activity inside the brain using superconducting sensors placed outside of the head. MEG provides very high temporal resolution and an excellent signal-to-noise ratio, making it useful for detecting subtle changes in neuronal firing patterns associated with BCI tasks. However, MEG systems are expensive and require specialized hardware not usually found outside research laboratories or hospitals. MEG’s key issues are described below:

- Good signal-to-noise ratio: MEG signals are relatively strong and easy to detect reliably, making them beneficial for BCI applications;
- High cost of equipment: the cost of equipment necessary for MEG is high, limiting its practicality in many settings;
- Limited spatial resolution: the spatial resolution of MEG is limited compared to other imaging technologies such as EEG, making it difficult to accurately map brain activity patterns with a single scan;
- Long acquisition times: the data acquisition times for MEG can be quite long, making it difficult to measure dynamic processes such as those involved in motor control tasks used in BCI systems;
- Head motion artifacts: head motion artifacts can significantly interfere with the accuracy of the recorded signal and lead to false positives or negatives, which could confuse the user’s experience with the system or even cause harm if medical decisions were made based on incorrect information from an artifactually contaminated signal.

ECoG involves placing electrodes directly onto the surface of the cortex during neurosurgery procedures such as epilepsy treatment or tumor removal operations. ECoG has excellent temporal and spatial resolutions due to its direct contact with cortical neurons; however, this comes at a cost—invasive surgery carries risks such as infection or bleeding, which may outweigh any potential benefits from using ECoG for BCI applications. Key issues with the ECoG platform are listed below:

- Signal acquisition: ECoG signals have relatively low amplitudes and might contain a certain degree of noise; therefore, reliable signal acquisition is essential for successful BCI applications;
- Data interpretation: properly interpreting the data collected from ECoG recordings can be challenging due to the complexity of neural activity as well as the need to distinguish between different types of brain activity (e.g., motor vs. non-motor);
- Safety concerns: since ECoG involves implanting electrodes directly onto the surface of the brain, there are potential safety risks that must be taken into consideration when designing an ECoG-based BCI system;
- Ethical considerations: the ethical implications associated with using invasive technology such as ECoG must also be considered when developing a BCI system for clinical use or research purposes.

Pros and cons for each technology are presented in Table 1.

The choice of a platform depends on several factors, such as the research goals, cost of equipment, patient comfort level, etc. EEG is widely used due to its low cost and portability. It has good temporal resolution but lacks spatial resolution, making it less suitable for some applications. fNIRS offers a non-invasive option with higher spatial resolution than EEG but lower temporal resolution. MEG offers excellent temporal and spatial resolutions with high accuracy; however, it comes at a much higher cost compared to other platforms. ECoG provides very high temporal and spatial resolutions but requires invasive surgery for the implantation of electrodes, which limits its use to certain clinical scenarios.

EEG is the most commonly used BCI platform in practice because of its relatively low cost, easy setup, and portability compared to the other mentioned platforms. Additionally, EEG provides excellent temporal resolution and can detect brain signals with millisecond accuracy. This makes it an ideal choice for real-time feedback systems such as those used in motor imagery or P300 speller implementations. In addition, this technique does not require any invasive procedures or radiation exposure, so it can be safely used on a wide variety of users, including children or elderly people who may have difficulty tolerating more intrusive monitoring methods.

The choice of BCI platform should be based on the research goals and other considerations such as cost of equipment or patient comfort level, rather than assuming one platform is better than another in general terms.

## 3. Classical Paradigms in BCI Systems

The most common types of BCIs are based on classical paradigms such as P300, steady-state visual evoked potentials (SSVEP), and motor imagery (MI).

The P300 paradigm uses EEG recordings from the scalp electrodes to measure event-related potentials generated when a user recognizes an important stimulus in a series of stimuli presented on a computer screen. It is one of the oldest BCI paradigms, developed in the 1980s [66]. It uses electrical signals from the brain to detect when a user is focusing on a particular stimulus or event. When this happens, an EEG signal called the P300 wave appears around 300 milliseconds after the event occurs. This wave can be used to measure how interested or involved someone is in what they are seeing or experiencing, and it can be used as input for BCIs. When presented with multiple stimuli on a computer screen, users typically respond more quickly when they recognize one particular target stimulus among them. This reaction time difference generates an EEG signal known as the P300 waveform, which can then be detected using scalp electrodes placed over different regions of the head. By monitoring this signal over time, it is possible to detect which stimuli were recognized by the user and thus infer their intentions. The P300 paradigm has a wide range of applications, ranging from medical rehabilitation, helping disabled patients regain movement through robotic prosthetics, to assistive communication, helping those who cannot speak communicate via text messages, security authentication, verifying identity without needing passwords, gaming, enhancing interactive experiences through mental commands, and cognitive assessment, evaluating patients mental states such as attention span and fatigue levels.

Steady-state visual evoked potentials (SSVEPs) utilize flicker frequencies, which present monitors generate periodic EEG patterns that correlate directly with the user’s gaze direction towards specific target screens [67]. By presenting rapidly changing colored squares in various locations of the display monitor, participants were instructed to focus their gaze onto each square in order to elicit a unique neural signature corresponding to the frequency of light being emitted from the object, thus allowing researchers to accurately track their eye movements around the environment without the need for any additional hardware equipment, such as cameras, or tracking their head movements manually. Furthermore, because flickering lights tend to remain visible longer than typical flashes, human eyes become accustomed to their frequency, easily reducing the possibility of errors arising from distractions outside the scope of the experimenter’s control while also increasing the maximum allowable response speed significantly compared to alternative methods such as P300. SSVEPs prove highly useful in a diverse range of fields, particularly ones involving virtual reality simulations, industrial robotics, advanced gaming technologies, artificial intelligence research, automated surveillance, biological engineering, etc. All benefiting greatly from improved response times, increased bandwidth, and offered protocols, along with the robustness factor brought to the table thanks to the simple yet reliable design structure operating behind the scenes. Other areas include, but are not limited to, medical diagnostics, psychological analysis, military operations, environmental monitoring, etc.

Motor imagery (MI) is a BCI paradigm where users are asked to imagine themselves performing certain movements without actually moving any body parts [68]. This type of system relies on EEG recordings from the scalp electrodes to measure changes in electrical activity within the brain that occur when a user imagines making specific motor actions. These changes can then be used to infer the intentions of the user and allow them to control external systems with just their thoughts. MI has been used for applications such as controlling wheelchairs, robotic arms, or other prosthetic devices, but it has also been applied in more novel ways, such as allowing people to play computer games using only their mind and even allowing paralyzed individuals to communicate by spelling out words letter-by-letter using mental commands alone. MI still has a wide range of uses today, including medical rehabilitation, helping patients regain lost limb functions through artificial prosthetics, gaming, enhancing interactive experiences through thought-controlled commands, communication aiding those who cannot speak via text messages, security authentication verifying identity without passwords, cognitive assessment evaluating patients mental states such as attention span fatigue levels, and finally, robotics, providing new ways to control machines remotely utilizing only brain power.

Advantages and disadvantages of these classical paradigms are summarized in Table 2.

## 4. BCI Signal Processing Techniques

A variety of signal processing techniques are employed when constructing a BCI system, including feature extraction algorithms such as independent component analysis (ICA), wavelet transformations, and autoregressive modeling; classification algorithms such as support vector machines (SVM); pattern recognition approaches such as hidden Markov models; machine learning models such as artificial neural networks; and optimization methods such as genetic algorithms or particle swarm optimization.

A key component of BCI’s signal processing is synchronization and asynchronization, which are methods used to establish a connection between the user’s brain signals and the computer system. Synchronization involves establishing an exact match between two signals, while asynchronization involves allowing for some variation in timing.

Synchronization is typically used when there is an exact time relationship required between two events or signals; this ensures that all data collected by one device is accurately transferred to another device at exactly the same moment it was acquired from its source. This type of synchronization requires precise timing control over both devices so that they remain synchronized throughout the duration of the data transfer. To achieve this level of accuracy, certain hardware components, such as clocks, must be employed to ensure accuracy over long periods of time without drift occurring due to environmental factors such as temperature changes or electrical interference from nearby devices. Synchronized data acquisition has been shown to improve signal detection accuracy compared to non-synchronized techniques since any temporal differences between acquisitions can be accounted for during analysis [69]. Additionally, synchronizing multiple channels allows EEG measures such as coherence values or event-related potentials (ERPs) to be measured across different electrode sites within each channel.

Asynchronous operations involve allowing some degree of variation in timing. Different from synchronized operations, where two events must happen simultaneously, asynchronous operations do not require absolute precision but rather permit some leeway when it comes to timing discrepancies [70,71]. This type of operation may also include elements such as buffering, which helps reduce latency issues associated with transmitting large amounts of data quickly across networks. Asynchronous methods have been shown to be effective for reducing false alarm rates in BCI applications since they allow more flexibility when dealing with erroneous inputs caused by noise contamination. They also provide better scalability than synchronous approaches since they do not need additional hardware components such as clocks, which can increase cost and complexity. However, asynchronous methods tend not to perform well in situations where real-time responses are essential, thus limiting their usage mainly to offline applications only.

Both synchronous and asynchronous techniques have been successfully applied to various types of BCIs, including motor imagery-based systems, P300 spellers, hybrid systems combining both EEG and EMG features, etc.

Each BCI signal processing technique has its own advantages depending on the application’s specific requirements, but all aim to accurately interpret user input from raw sensory data collected by sensors attached directly to the user’s head or body.

### 4.1. ICA Use in BCI Systems

Independent component analysis (ICA) is a powerful statistical technique used to identify and separate independent sources of information from data streams. It can be used in brain–computer interface (BCI) systems to improve the accuracy of EEG signal processing by separating useful signals from noise or artifacts. ICA helps extract meaningful information, such as event-related potentials, which are then further processed for better understanding brain activity patterns during tasks. Additionally, ICA can be used for artifact removal purposes, allowing BCI applications to reduce false-positive detections caused by movement artifacts or other interference signals.

Independent Component Analysis (ICA) has been employed to decompose multichannel datasets into independent components based on certain assumptions [72]:

- The number of independent components must not exceed the number of electrodes used in recording EEG signals;
- Neuronal and artifact sources are considered to be linearly mixed yet independent from each other;
- A negligible signal propagation delay is assumed between brain sources and electrodes.

The objective of ICA is to identify a linear projection that maximizes mutual independence, which can be mathematically expressed as:

x(k) = As(k), k = 1, 2, 3, …, N,(1)

where: $x\left(k\right)\in {ℜ}^{M\times 1}$ are recorded EEG signals, $s\left(k\right)\in {ℜ}^{M\times 1}$ are corresponding independent components, $A\left(k\right)\in {ℜ}^{M\times M}$ is unknown M full rank mixing matrix, k—discrete time, and M is the number of electrodes.

Independent components can be represented as

(2) $$s_{i}\left(k\right)=w_{i}^{T}x\left(k\right),\;k=1,\;2,\;3,\;\ldots ,\;N,$$

where i is the electrode ordinal number (i = 1, 2, 3, …, M), $w_{i}$ is the column vector.

After evaluating each w_i_, the independent components can be calculated [73] as:

(3) $$s_{i}\left(k\right)=\mathrm{Wx}\left(k\right),\;W\approx A^{-1}.$$

The ICA models utilized by algorithms such as Infomax, JADE, FastICA, and RADICAL assume that the sources of independent components are either non-Gaussian or a single source is Gaussian. These approaches do not consider the temporal structure of the signal or extract components with a Gaussian distribution. To address this limitation, time series-based ICA models implemented in TDSEP/SOBI, and AMUSE can select independent components with a Gaussian distribution [74]. Extended-Infomax ICA has the capacity to remove both super-gaussian artifacts (eye blinks) and sub-gaussian signals (power noise interference), whereas regular Infomax ICA is limited to the removal of super-gaussian artifacts only.

ICA algorithms have been found to have numerous drawbacks, including ambiguity regarding the origin of sources, uncertainty with regards to component dispersion, and dependence on data set size; in particular, ICA algorithms are characterized by inadequate performance when operating on small data sets [75].

### 4.2. Wavelet Transformations and Autoregressive Modeling in BCI

Wavelet transformations and autoregressive modeling are used in BCI systems to identify patterns in brain signals. The wavelet transform can be used to decompose the EEG data into its frequency components, allowing for an analysis of different frequencies associated with cognitive tasks. Autoregressive modeling uses a linear regression model to estimate the current value of a signal based on past values, which can then be used in conjunction with classification algorithms such as support vector machines or neural networks to detect subtle changes in EEG signals that may indicate certain mental states. Together, these two methods provide powerful tools for understanding how our brains interact with technology and allow us to develop better BCI systems that respond more accurately and effectively to user inputs.

The wavelet transform (WT) is a one-dimensional technique that decomposes an electroencephalographic signal into a set of coefficients representing its similarity with the waveform of a parent wavelet at certain scales. This transformation can be expressed mathematically as selecting a subset of scales (j) and time shifts (k) of the parent wavelet (t):

(4) $$\Psi_{j,k}\left(t\right)=2^{\frac{j}{2}}\Psi \left(2^{j}t-k\right),$$

where j and k are integers.

The Discrete Wavelet Transform (DWT) is a method for calculating the scaling and detail components of a waveform by low- and high-frequency filtering, where the waveform is discretely sampled in time.

The use of DWT for artifact removal is often achieved through thresholding the decomposed coefficients and reconstructing the remaining signal components, channel by channel [76]. A disadvantage of DWT is that it cannot completely remove artifacts when the spectral properties of the signal being studied overlap with those of the artifacts.

The temporal dispersion, which is the main DWT disadvantage, is eliminated in the stationary wavelet transform (SWT) algorithm, which does not involve downsampling. SWT has been shown to enable the tracing of changes in the harmonic components of EEG signals over time [77].

Regression analysis has been demonstrated to be relatively straightforward to use; however, specific assumptions must be adhered to in order for accurate results [78]. (1) The native EEG signal is a combination of genuine neuronal activity and extraneous activity (an artifact); (2) true neuronal activity and extraneous activity of the EEG signal are uncorrelated; (3) artifacts should not contain components related to brain activity; otherwise, they may be lost during artifact removal.

Regression algorithms necessitate exogenous reference channels (e.g., EOG, ECG) for artifact detection; however, it is challenging to identify the most suitable reference signal for myographic and other non-biological artifacts, hence constricting the use of regression methods for artifact removal.

The use of temporal auto-tuning for EEG signal activity has proven to be an effective method for detecting artifacts among evoked brain activity, yet it does not account for background activity. Regression methods can identify oculographic artifacts but are limited in their ability to reduce bilateral contamination between EEG and EOG [79]. To address this issue, low-pass filtering before applying Bayesian adaptive regression splines has been proposed as a solution [80], as the high-frequency content of the recorded EOG typically refers to neural activity, which can be filtered out to significantly reduce bidirectional pollution effects.

The literature overview showed a lack of consensus on the best low-pass filtering of EOG signals. In contrast, some researchers have suggested that all frequency bands in the EOG signal are associated with neuronal activity [81].

Despite their shortcomings, regression methods are regarded as the “gold standard” for evaluating the effectiveness of new artifact detection algorithms.

### 4.3. SVM in BCI Systems

SVMs are used in BCI systems for classification tasks, such as recognizing patterns in EEG signals. SVMs can be used to identify features from the raw EEG data and classify them into different categories or classes. For example, an SVM may be used to distinguish between different brain states, such as sleep states or mental workload levels. Additionally, SVMs can be trained on specific tasks, such as imagined hand movement recognition and motor imagery-based BCI control. The high accuracy of SVM algorithms makes them well suited for use in BCI applications where accurate classification is essential.

SVM, proposed by V. Vapnik and A. Chervonenkis [82], is a method of linear classification that divides the sample into classes using an optimal separating hyperplane with the following general equation form:

(5) f(x)=[ω,φ(x)]+b,

where $\omega =\overset{N}{\underset{i=1}{\sum }}\lambda_{i}y_{i}\varphi_{i}\left(x_{i}\right)$, and the coefficients $\lambda_{i}$ depend on the vector of labels $y_{i}$, and on the scalar products $\varphi_{i}\left(x_{i}\right)$. To find the decision function, knowledge of these scalar products is necessary. Data transformations are determined by a kernel function: K(x,y)=[φ(x), φ(y)].

In 1992, a non-linear Support Vector Machine (SVM) classification method was proposed by utilizing a non-linear kernel function [82,83]. This approach enables the search for the optimal separating hyperplane in the transformed feature space. The radial Gaussian basis function can be used as a kernel function:

(6) $$K\left(x_{i},x_{j}\right)=\exp\left(-\gamma \|x_{i}-x_{j}{\|}^{2}\right),\;\mathrm{for}\;\gamma >0.$$

The classifier is divided into two stages: training and testing. The data are first split into a training set with assigned class labels and a test set without them. During the first stage, the classifier builds a model based on the training sample and divides it into given classes. In the second stage, the constructed model is tested by feeding in the test sample (without class labels) to determine if EEG patterns belong to possible classes. The classification accuracy (the ratio of correctly determined samples over the total number of samples expressed as a percentage) then measures how effective the classifier was.

### 4.4. HMM’s for BCI

Hidden Markov models (HMMs) are used in BCI systems to recognize user intentions based on their brain signals. HMMs can be trained to detect patterns in EEG signals, such as movement intent or mental arithmetic tasks, and classify them into different classes. The model is then able to predict the user’s intention from the observed data and use it to control a computer system or robotic device. In addition, HMMs can be used for decoding motor imagery activities, which allow users with severe disabilities to interact with computers using only their minds.

The HMM method, based on the Bayesian posterior probability maximization approach, has been successfully employed to classify time series, with states at any given time t being affected by states at the previous time (t − 1). This technique has shown its utility in resolving speech recognition problems and is widely used for signal analysis, classification, modeling, and control purposes [84].

The solution of three fundamental problems is required to construct a Hidden Markov Model (HMM) for the given sequence of observed states:

1. The evaluation problem can be stated as follows: Given an HMM with transition probabilities a_ij_ and b_jk_, determine the probability that a particular sequence of visible states (VT) was generated by this model.
2. Decoding problem. Given an HMM and a set of observations (V^T^), we need to determine the most probable sequence of hidden states ω^T^ that result in these observations.
3. The learning problem. Given an enlarged structure of the model with a specified number of states and visible states but without knowledge of transition probabilities a_ij_ and b_jk_, learning can be performed by determining the most plausible model from a training sample of visible states.

Problems 1 and 2 are solved at the decoding stage using forward procedures or Viterbi algorithms [84]. Problem 3 is tackled in the learning process by employing an iterative procedure for finding a local maximum, such as the Baum–Welch algorithm [84], or utilizing global optimization algorithms, e.g., simulated annealing [85].

HMMs can be utilized in BCIs as probabilistic automata that calculate the likelihood of a given sequence of feature vectors. Each state of the automaton models the probability distribution for observing a particular feature vector, with Gaussian models being commonly used in BCI applications [83].

The use of HMMs for classifying time series has been demonstrated to be effective due to their inherent nature. As the EEG contains distinct features that can be distinguished in the temporal domain, the HMM method was utilized for EEG classification in BCI [86,87,88]. However, HMMs have not seen widespread application in BCI development yet, despite the relatively high success rates reported by known studies. One major obstacle is the necessity of identifying an invariable set of observable states related to an event, which may prove difficult when analyzing EEG signals; for example, while studying induced desynchronization as reported in [80] across different channels. If signals associated with close localized events and similar dynamics are being analyzed, it becomes hard to identify stable observable states; requiring instead the identification of process attractors related only to classified events without including background nervous system activity. A further exploration into EEG signals and neurophysiological brain functioning fundamentals could possibly provide such an opportunity, thus increasing the relevance of using HMMs significantly.

### 4.5. Neural Network Algorithms for BCI Systems

Machine learning models such as artificial neural networks (ANNs) are used in BCI systems to help interpret and classify the brain signals that they receive [89,90,91]. By utilizing ANNs, a BCI system can be trained to recognize patterns in EEG data, which can then be used to detect changes in states of consciousness or other types of mental activities. This allows BCI systems to respond more accurately and quickly than traditional methods would allow. Additionally, by using ANNs for pattern recognition tasks, it is possible for a BCI system to adapt over time as new patterns emerge from the EEG data. This ability makes them especially useful for applications involving long-term monitoring of patients with neurological disorders such as epilepsy or dementia.

Convolutional neural networks (CNN) are most commonly used for this task. CNNs have been proposed by LeCun [92] as a type of artificial neural network architecture for efficient pattern recognition in images. CNNs are composed of convolutional layers and pooling layers, which enable the extraction of features from input data while reducing the amount of processed information and preserving task-specific information.

**Figure 4:**
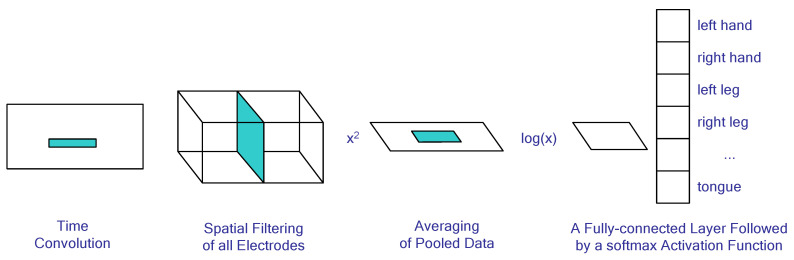
The CNN architecture in BCI analysis.

When working with EEG signals, convolutional networks can be used to reduce the problem of image classification by feeding spectrograms into their inputs. Alternatively, one may use an adaptation of the FBCSP method as an input architecture; for example, ShallowNet is described in [93] and illustrated in Figure 4. The layers composing this architecture and their respective functions are detailed as follows: A 1 × 25 time convolution is implemented to highlight characteristic peaks in the signal, followed by a spatial filtering of all electrodes similar to that of the FBCSP algorithm. Subsequently, an element-wise squaring is performed on the matrix values before proceeding with a 1 × 75 windowed time pooling operation, wherein the average value of each window element is taken. A natural logarithm transformation then follows for each element, which is equivalent to calculating the logarithm of signal dispersion as seen in FBCSP. Finally, these features are classified by combining fully connected and softmax layers.

Deep learning methods are reliant on the amount of data available; as more data is used, better generalization occurs. To counter this issue, augmentation methods have been utilized.

### 4.6. Genetic Algorithms and Particle Swarm Optimization in BCI

Genetic algorithms (GA) and particle swarm optimization (PSO) are optimization methods that can be used to optimize or tune the parameters of a BCI system. GA is an evolutionary algorithm that uses concepts such as mutation, crossover, and selection to find optimal solutions. PSO is an iterative algorithm inspired by social behavior in which particles move around in search space with velocities that are influenced by their own best position and the global best position found so far. Both algorithms can be used to automatically adjust model parameters within a BCI system, thereby improving its performance. For example, they could be used to optimize feature extraction techniques for EEG signals or adaptively select appropriate stimuli for brain–computer interfaces based on user feedback.

GAs use the principles of natural selection and genetics to find solutions to complex optimization problems. They are used for tasks such as finding the optimal parameters for a machine learning model or finding the shortest route from one point to another in a network. GAs have been increasingly used in BCI systems as they offer an effective way to optimize BCI performance by automatically searching through a large space of potential parameter values and selecting those that yield better results [94,95,96,97,98]. As advantages of Gas could be mentioned, their robustness. Gas are able to handle noise, nonlinearities, and outliers without much difficulty. This makes them particularly well suited for BCIs, where there is often considerable uncertainty due to biological variability between users or individuals with different levels of expertise using the system. Another advantage is their efficiency. Gas can be implemented quickly and easily compared to other optimization techniques such as gradient descent or simulated annealing, making them ideal for real-time applications such as online control systems where speed is essential. Finally, due to their flexibility, GAs can be applied to many different types of problems, including classification, regression, clustering, etc., making them applicable across many different domains within BCI research.

On the other hand, these methods are known for their computational complexity. While GAs provide efficient solutions compared to other methods, they require more computational resources than some alternative approaches such as grid search or random search, which may limit their applicability on certain platforms with limited memory or computing power available, e.g., embedded devices used in mobile applications. In addition, they are limited in their interpretability. Due to their nature as black box models, it can be difficult to interpret why certain decisions were made by the algorithm, which could lead to issues when trying to debug any errors during the development stages or if unexpected behavior occurs while using the system live.

PSO in BCI has been used for feature selection, parameter tuning, and model selection. Its strong sides are [99,100,101,102]:

- PSO is simpler than other optimizers since it does not require costly derivatives or linear algebra operations;
- PSO can be used with any type of problem formulation, such as discrete, continuous, constrained, or unconstrained optimization problems;
- PSO can find solutions faster compared to traditional algorithms because it uses parallel computing techniques that allow multiple particles to explore the search space simultaneously and cooperatively;
- The algorithm is easy to implement due to its simple structure and few parameters to adjust during its execution process.
- It does not require an initial guess from the user and thus can be useful in cases where one may not know what kind of solution they are looking for.

On the other hand, PSO’s downsides are the following:

- The results obtained by using this method depend on the choice of parameters such as inertia weight, cognition factor, social factor, etc., so if these values are set too high or too low, then the result will also suffer accordingly.
- It may take more time than other methods since many iterations need to be done until a good solution is found;
- Some features may remain unexplored due to a lack of exploration strategies implemented in some versions of PSO algorithms, resulting in sub-optimal solutions being returned instead of optimal ones.

### 4.7. BCI Datasets and Benchmarking

In order to assess the performance and accuracy of different BCI techniques and methods for designing these interfaces, BCI datasets are typically used. These datasets are collections of data gathered from individuals using BCI systems and provided either by research organizations, BCI manufacturers, or individual researchers. Commonly used BCI datasets include NeuroSky Mindwave [103], Emotiv EPOC+ [104,105], OpenBCI Ganglion [106], Graz University EEG Motor Imagery Database [107], PhysioNet EEG Motor Movement/Imagery Dataset [108], etc. These datasets provide a variety of recordings, including raw EEG data as well as preprocessed information such as event-related potentials (ERPs).

The NeuroSky Mindwave dataset consists of recordings made during various cognitive tasks such as mental arithmetic and memory recall, while the Emotiv EPOC+ dataset contains recordings taken during emotional recognition tasks. The OpenBCI Ganglion dataset includes both resting state and motor imagery recordings, while the Graz University EEG motor imagery database provides detailed information on motor imagery-related activities performed by subjects in an experimental setting. Finally, PhysioNet’s EEG motor movement/imagery dataset offers multiple types of motor imagery tasks along with corresponding scores indicating how accurately each task was performed by participants.

Another widely used BCI benchmark dataset is described in [109,110,111]. These datasets were initially used in BCI research competitions in the early 2000s, and could now be used to assess the performance and accuracy of novel BCI techniques and methods. These datasets typically consist of EEG signals recorded by participants as they perform various tasks. The most commonly used BCI benchmark datasets include [109,110,111]:

- BCI Competition IV Dataset 2a: This dataset consists of EEG and EOG recordings from nine subjects performing motor imagery tasks such as left/right hand or foot movement, imagining a circle or a line, and other more complex movements;
- The BCI Competition IV Dataset 2b: This dataset consists of EEG recordings from nine subjects performing motor imagery tasks while a visual cue was presented at different time points during the task;
- The BCI Competition IV Dataset 3: This dataset consists of MEG recordings from two subjects performing motor imagery tasks such as wrist movement in different directions;
- The BCI Competition IV Dataset 4: This dataset contains ECoG recordings from three subjects performing motor imagery tasks such as finger movement acquired with a data glove;
- The OpenMIIR Dataset [112]: This dataset includes EEG recordings from 20 healthy volunteers who were asked to imagine either moving their hands, feet, tongue, or eyes in order to control a virtual avatar on screen by using their thoughts alone;
- The High-Gamma Dataset (HGD) [113,114]: This dataset is composed of high-gamma-power EEG signals that can be used for studying the neural correlates associated with visual perception and memory encoding processes in humans using machine learning algorithms.

Thus, existing BCI datasets allow one to assess novel BCI techniques and algorithms, helping to improve their performance and accuracy.

### 4.8. Noise and Environmental Disturbances Impact on BCI Systems

As with any form of communication, noise and environmental disturbances can greatly reduce the efficiency of BCI systems. The most common type of external disturbance encountered by BCI systems is acoustic noise. This includes any sound produced by people or machines in close proximity to a BCI system user, such as conversations, typing noises, etc., that may interfere with accurate EEG signal acquisition or processing. Studies have shown that even low levels of background noise can significantly reduce accuracy when trying to distinguish different mental states (e.g., attention vs. relaxation). Furthermore, some studies also suggest that certain frequencies can be more disruptive than others depending on their similarity to those present in EEG signals; therefore, it is important to identify these frequencies prior to using a BCI system in order to minimize interference from external sources [115].

In addition to acoustic sources of interference, there are also numerous types of electromagnetic fields present in everyday life that may interfere with the proper functioning of a BCI system due to its reliance on electrical signals generated by neurons within the brain [116]. Common sources include power lines, radio waves emitted from cell phones or Wi-Fi routers, etc., all of which could potentially disrupt neural activity recorded via EEG electrodes, thus reducing overall accuracy when detecting specific mental states or commands given by users [117]. Therefore, it is essential that adequate shielding measures are taken during the design and implementation stages so as not to compromise performance due to unwanted outside influences [118].

Noise and environmental disturbances can severely affect the efficacy of BCI systems if left unchecked; however, appropriate measures taken during the development stages concerning both acoustic/mechanical interferences as well as electromagnetic ones should help ensure maximum efficiency when using such technologies going forward into future applications involving human–machine interactions.

## 5. Applications

BCIs have been proposed for use in many fields, including medicine, neuroscience research, education/training environments, human–computer interaction, and even gaming/entertainment applications where users can control virtual objects using only their thoughts without any physical movement required. In addition to these more traditional uses, there is also ongoing work exploring new areas such as thought-controlled wheelchairs, which allow disabled people greater freedom of mobility without relying on manual controls; prosthetic devices enabling amputees to have better manipulation capabilities than ever before; communication aids designed specifically for people suffering from severe speech impairments; remote monitoring systems that track vital signs while allowing patients greater independence at home rather than having them stay confined in hospitals; and even mind-controlled drones. The possibilities seem endless when considering what could be achieved if we were able to understand our brains better, so let us take a look at some examples where this technology has already made an impact.

### 5.1. Neuroprosthetics

BCIs are being used to create neuroprosthetic devices, which allow people with physical disabilities to control external devices such as wheelchairs and robotic arms using their own brain signals. For example, the BrainGate neural interface system is a device that can be implanted in the brain to record electrical activity from neurons and translate it into commands for controlling external devices.

The use of BCIs in neuroprosthetics is a rapidly growing field, with potential applications ranging from restoring communication to those who have lost it due to injury or illness to providing enhanced control of prosthetic limbs. BCI technology has been used for decades in the medical sector but only recently began being applied to the development of neuroprostheses [9,12,119,120].

One example of BCI technology being used in neuroprosthetics is brain-controlled robotic arms and hands [121,122,123,124,125]. These are designed to allow users with spinal cord injuries or amputations to move their prosthetic limb by simply thinking about it, rather than having to manually control it using switches or joysticks. This type of device can also be used as an assistive tool for people with limited motor skills, such as stroke victims or those suffering from degenerative diseases such as ALS (amyotrophic lateral sclerosis). By interpreting electrical signals produced by neurons in the user’s brain, these devices can accurately predict what action they should take when given input from the user, allowing them greater independence and mobility.

Another application for BCI technology within neuroprosthetics is its use in restoring communication capabilities for those unable to speak due to paralysis caused by conditions such as ALS, stroke, or traumatic brain injury [126,127]. In this case, electrodes placed on the scalp detect electrical activity produced by neurons that would normally be associated with speech production and then translate this into words spoken through a computerized voice synthesizer. This allows individuals who cannot physically produce sound themselves to still communicate their thoughts and feelings without relying solely on writing them down or typing out messages using eye-tracking software programs—enabling them much more freedom than before.

Finally, neural implants are another form of BCI technology currently being explored within the realm of neuroprosthetics research—particularly as part of “neurohybrid” systems combining both biological components (such as nerves) and artificial ones (such as microprocessors) [121,128,129]. Neural implants involve surgically implanting electrodes directly into areas responsible for controlling movement so that they can receive direct commands from neuronal activity generated there instead—potentially resulting in even faster response times than traditional forms of BCIs, which rely on detecting signals transmitted through scalp electrodes alone.

Overall, BCI technology is an exciting new field with a wide range of potential applications within the realm of neuroprosthetics—from restoring communication capabilities to providing enhanced control over prosthetic limbs and beyond. As research continues to progress in this area, it can be expected that further advancements will be made that will allow individuals with disabilities greater independence and mobility than ever before.

### 5.2. Communication

BCI technology is also being used to develop new ways of communicating for people who have lost the ability to speak or write due to paralysis or other conditions. For example, BCI systems can be used to detect intentions from users’ brain signals and then convert them into text messages or even speech output through computer algorithms [14,130].

BCIs have become increasingly popular in recent years as a way to enable communication between humans and machines. BCIs are devices that measure brain activity, such as electrical signals from the brain, and then use this information to control external objects or systems. BCIs can be used for a variety of applications, including controlling prosthetics, medical diagnosis, rehabilitation therapy, gaming, robotics control, and even communication.

This type of research is promising as it could be used to help people with disabilities who cannot communicate verbally or physically due to paralysis or other conditions.

Other studies have looked into how BCI technology can be used for more complex forms of communication, such as typing on a computer keyboard or giving speech commands via voice recognition software [131,132,133,134,135,136]. These types of applications could prove useful for helping individuals with severe motor impairments regain some level of independence when communicating with others. Additionally, there have also been attempts at developing interfaces that allow users to generate language through thought alone using EEG recordings. While these technologies are still relatively new and require further development before they can be widely adopted, they represent an exciting potential future application for augmenting human-machine interaction via BCI technology.

Overall, BCI technology has the potential to revolutionize communication as we know it. While there is still a lot of research and development needed before this technology can be widely adopted, the potential for enabling individuals with disabilities to communicate more effectively or even generate language through thought alone is an exciting prospect.

### 5.3. Gaming

BCIs are increasingly being used in gaming applications where players can interact with virtual environments using only their thoughts instead of traditional controllers such as keyboards and joysticks [137,138,139,140,141,142].

One example of a game that utilizes BCI is MindRDR, developed by the London-based startup This Place [143]. The game uses EEG sensors to measure players’ emotional responses while playing. Players use their mental focus or concentration levels to control the direction and speed of an avatar on screen. As players become more emotionally engaged with the game, their avatar will move faster and farther across the screen than if they were not as focused or engaged with it.

Another example of a BCI-enabled video game is Brain Wars from NeuroSky Inc., which allows players to compete against each other using EEG headsets to measure brainwaves associated with concentration levels during gameplay [144,145]. Players must concentrate hard enough so that their brain waves reach certain thresholds in order to be able to progress through different levels in the game.

In addition, there are several research projects underway exploring how BCIs can be used for virtual reality gaming experiences [146,147].

Overall, BCIs offer great potential when it comes to enhancing gameplay experiences by providing gamers with new ways of experiencing games beyond just pressing buttons on controllers or keyboards—allowing them instead to tap into emotions and thought processes unique to themselves! With further advancements in technology, BCIs could become a major part of the gaming industry in the future.

### 5.4. Education

BCIs are being used to enhance the learning experience by providing real-time feedback about students’ cognitive states and helping them focus better on their studies.

This technology has been used in various ways for educational purposes, ranging from helping students with special needs learn how to control their movements and communicate effectively to providing more immersive learning experiences for all learners [148,149,150,151].

Research on the use of BCI in education has shown positive results when it comes to improving student engagement and motivation. For example, one study found that using BCI-based games improved cognitive skills among students who had difficulty paying attention during traditional classroom activities. Additionally, research suggests that BCI can be used as an effective tool for teaching abstract concepts such as mathematics or foreign languages by allowing users to directly experience the material instead of relying solely on verbal instruction.

Furthermore, studies have demonstrated that the use of BCIs can reduce stress levels among students by providing them with a more natural way of interacting with computers than conventional input devices such as keyboards or mice. Finally, research indicates that BCIs may provide new opportunities for personalized learning since they allow teachers to tailor lesson plans according to individual students strengths and weaknesses based on real-time feedback from brain activity data.

Overall, research suggests that BCI technology has the potential to revolutionize education by providing more engaging and immersive learning experiences for students of all ages.

### 5.5. Mental Health

BCI technology is also being explored as a potential treatment for mental health conditions such as depression, anxiety, and addiction by allowing clinicians to monitor patients’ brain activity in real time and provide targeted interventions when required [152,153,154,155].

In mental health care, BCI can be used to assess cognitive processes such as attention and memory; detect changes in emotional states; monitor progress in therapy; measure levels of stress or relaxation; provide feedback during biofeedback exercises; diagnose neurological disorders such as Alzheimer’s disease or Parkinson’s disease; improve motor skills after stroke or traumatic brain injury (TBI); help people suffering from depression manage their symptoms through self-regulation techniques; reduce anxiety associated with public speaking; and more. In addition, BCIs have been shown to be effective tools for helping patients develop better coping skills when dealing with difficult emotions such as anger or fear.

Research suggests that BCI could potentially revolutionize how we approach mental healthcare by allowing us to quickly identify psychological problems at an early stage and intervene before they become serious issues. For example, some research has suggested that EEG-based BCIs may be able to detect subtle signs of distress related to depression that would not normally be picked up by traditional methods of assessment such as questionnaires or interviews alone. Other research has demonstrated how portable EEG systems can be used in real-time settings outside of a clinical environment, providing clinicians with valuable information about the patient’s condition without having the patient physically present in front of them.

### 5.6. Sleep Medicine

Recently, there has been an increasing interest in BCI’s potential application for sleep medicine applications, such as improving sleep quality and diagnosing different psychiatric and neurodegenerative diseases by analyzing sleeping stages [156,157,158,159,160,161,162]. The use of BCIs could help individuals track their sleeping patterns more accurately than traditional methods.

One way this technology can be utilized is through EEG-based BCI systems, which measure electrical activity in the brain during different stages of sleep. These devices are able to detect changes in neural oscillations associated with rapid eye movement (REM) and non-REM sleep cycles, allowing accurate tracking of the user’s progress throughout the night.

Another promising application for sleep BCIs is treating various types of insomnia by providing targeted neurostimulation therapies such as transcranial alternating current stimulation (tACS). tACS uses low-intensity electric currents delivered directly into specific areas of the brain believed to regulate wakefulness/sleep cycles, thus helping reset abnormal rhythms associated with poor sleeping habits or disruptions caused by stressors such as jet lag or shift work schedules.

Furthermore, research suggests that combining tACS with cognitive behavioral therapy might produce better results compared to either treatment alone when it comes to managing chronic sleeplessness conditions such as primary insomnia disorder or obstructive sleep apnea syndrome (OSAS).

Sleep brain computer interfaces offer great potential for improving our understanding of how we process information during restful states while also enabling more effective treatments for common problems related to the lack/disruption of adequate shut-eye—from mild cases involving occasional difficulty falling asleep all the way up to severe clinical disorders such as OSAS. As these technologies continue to advance over time, we should see further improvements both in terms of accuracy when monitoring physiological parameters related to slumber and in terms of efficacy when delivering personalized interventions meant to optimize one’s overall wellbeing.

Overall, there is still much work needed before we can fully understand the potential benefits offered by this exciting new technology, but initial findings suggest it could dramatically improve our understanding of mental illness while offering patients access to more personalized treatments tailored specifically for their individual needs.

## 6. Conclusions

The paper presented the current state-of-the-art in brain-computer interface technologies. Main platforms used for BCI data collection, such as EEG, fNIRS, MEG, and ECoG, were reviewed, and their pros and cons were singled out. It was concluded that the choice of a platform depends on the research goals, cost of equipment, patient comfort level, etc., while it is not correct to say that one of the platforms is better than others in general.

The most widely used BCI signal processing techniques, such as ICA, wavelet transformation, SVM, hidden Makrov models, machine learning, and genetic algorithms, were reviewed. Brief principles of their operation and main application areas are highlighted.

Finally, the main BCI system application areas, such as neuroprosthetics, communication, gaming, education, and mental health care, were reviewed.

It was highlighted that BCI offers tremendous potential opportunities across multiple domains—both existing ones, such as medical treatment and monitoring, and entirely novel concepts, such as controlling drones via thought alone. There is still much progress needed, however, before these ideas become realities—further technological developments must continue alongside increased understanding about how our brains actually function so that reliable interactions between humans and machines can be established and maintained over time safely and effectively.

## Figures and Tables

**Table 1:** BCI platforms pros and cons.

Platform	Pros	Cons
EEG	- Low cost. - Portable, noninvasive, and easy to use. - Can provide a high temporal resolution of brain activity.	- Low spatial resolution due to the wide distribution of electrodes on the scalp; - Susceptible to artifacts from eye movements, muscle contractions, etc., which can make data interpretation difficult or impossible in some cases;
fNIRS	- Noninvasive technology that is relatively portable and low cost compared to other BCI platforms. - Highly sensitive and able to detect changes in oxygenated blood levels at different depths within the brain tissue with good accuracy when properly calibrated.	- Lower temporal resolution than EEG or MEG systems due to its reliance on hemodynamic responses rather than electrical signals from neurons directly. - Not suitable for measuring deep brain structures because it relies on light transmission through skull bones, which may be impeded by thicker skulls or dense bone structures such as those found in elderly individuals or children under 5 years old, respectively.
MEG	- Extremely high temporal resolution as well as excellent source localization capabilities.	- Expensive equipment is required for setup and operation. - Requires highly trained personnel for proper calibration and signal processing. - Susceptible to environmental interference such as electromagnetic fields generated by nearby electronics such as cell phones or computers, which can distort readings if not properly shielded from these sources before measurements are taken.
ECoG	- High spatial accuracy due to direct contact with cortical surface - Excellent signal quality with much less noise than EEG recordings - High temporal resolution comparable to MEG systems	- Invasive procedure requiring surgery for the implantation of electrodes - Risk of infection associated with surgical procedures - Risk of tissue damage due to the implantation of electrodes and/or surgical procedures

**Table 2:** BCI classical paradigms pros and cons.

Paradigm	Pros	Cons
P300	- High accuracy rate; can achieve recognition rates up to 99%; - Since no extra hardware or special training is required beyond wearing EEG sensors, it is easy for anyone with minimal technical knowledge to use it effectively; - Plenty opportunity for optimization due to its long history within BCI research.	- Slow response times due to the large number of trials needed before accurate responses can be obtained; - Susceptibility noise interference affecting the quality of data collected from participants brains; - Low information transfer rates, caused largely by the limited number of channels available to record signals.
SSVEP	- Simplicity of a setup process, not needing anything beyond a traditional PC monitor to create an effective interface, along with the general robustness protocol itself capable of detecting small variations in input; - Thanks to much faster response speeds than many alternatives, SSVEPS offer higher bandwidth transmission values, meaning larger amounts of data can be sent between machine operators in a short period of time, which is important in real-time applications.	- Inability to account for any sudden changes in inputs under circumstances occurring outside the experimenter’s expectation of a result; - Lack of flexibility and adaptability, demanding certain predetermined conditions be met to ensure the proper functioning of the device; - Problem regarding the portability aspect since the calibration process requires considerable effort in order to setup the the system in the first place before actual testing begins while also taking quite a bit power to run continuously and maintain accuracy throughout the duration of the session, causing major hindrances in mobile implementations where size and weight matter most.
MI	- Flexibility, since no physical movement is required; it allows users with disabilities or limited mobility greater freedom in terms of how they interact with computers and other electronic devices. - Since EEG signals generated by MI are relatively easy to detect compared to other BCI paradigms such as SSVEP or P300, there is less need for complex signal processing algorithms, which makes implementation easier and faster overall; - Due its non-invasive nature, this method does not require costly hardware setups or any special training, thus keeping setup costs low while increasing accessibility for anyone wanting to use technology.	- Difficulty in distinguishing between actual and imagined motor tasks under the same conditions, leading to significant confusion errors; - Accuracy rate drops heavily depending on task complexity and the number of decisions needed to be made during a single session, meaning some applications may not be suitable given current limitations despite the potential advantages offered by the protocol itself; - Due reliance purely on internal processes such as imagination, fatigue becomes a major problem, especially for longer sessions, causing a drop performance over time and the need for additional breaks to recover properly before continuing operation, further reducing the effectiveness of the system as a whole.

## Data Availability

Not Applicable.

## References

[1] Mridha M.F., Das S.C., Kabir M.M., Lima A.A., Islam M.R., Watanobe Y. (2021). Brain-Computer Interface: Advancement and Challenges. Sensors.

[2] Mora-Cortes A., Manyakov N.V., Chumerin N., Van Hulle M.M. (2014). Language Model Applications to Spelling with Brain-Computer Interfaces. Sensors.

[3] Belwafi K., Gannouni S., Aboalsamh H. (2021). Embedded Brain Computer Interface: State-of-the-Art in Research. Sensors.

[4] Värbu K., Muhammad N., Muhammad Y. (2022). Past, Present, and Future of EEG-Based BCI Applications. Sensors.

[5] Siribunyaphat N., Punsawad Y. (2023). Brain–Computer Interface Based on Steady-State Visual Evoked Potential Using Quick-Response Code Pattern for Wheelchair Control. Sensors.

[6] Xie Y., Oniga S. (2023). Classification of Motor Imagery EEG Signals Based on Data Augmentation and Convolutional Neural Networks. Sensors.

[7] Tsiamalou A., Dardiotis E., Paterakis K., Fotakopoulos G., Liampas I., Sgantzos M., Siokas V., Brotis A.G. (2022). EEG in Neurorehabilitation: A Bibliometric Analysis and Content Review. Neurol. Int..

[8] Saichoo T., Boonbrahm P., Punsawad Y. (2022). Investigating User Proficiency of Motor Imagery for EEG-Based BCI System to Control Simulated Wheelchair. Sensors.

[9] Abdullah, Faye I., Islam M.R. (2022). EEG Channel Selection Techniques in Motor Imagery Applications: A Review and New Perspectives. Bioengineering.

[10] Gannouni S., Belwafi K., Al-Sulmi M.R., Al-Farhood M.D., Al-Obaid O.A., Al-Awadh A.M., Aboalsamh H., Belghith A. (2022). A Brain Controlled Command-Line Interface to Enhance the Accessibility of Severe Motor Disabled People to Personnel Computer. Brain Sci..

[11] Asanza V., Peláez E., Loayza F., Lorente-Leyva L.L., Peluffo-Ordóñez D.H. (2022). Identification of Lower-Limb Motor Tasks via Brain–Computer Interfaces: A Topical Overview. Sensors.

[12] Singh S.P., Mishra S., Gupta S., Padmanabhan P., Jia L., Colin T.K.A., Tsai Y.T., Kejia T., Sankarapillai P., Mohan A. (2023). Functional Mapping of the Brain for Brain–Computer Interfacing: A Review. Electronics.

[13] He Z., Li Z., Yang F., Wang L., Li J., Zhou C., Pan J. (2020). Advances in Multimodal Emotion Recognition Based on Brain–Computer Interfaces. Brain Sci..

[14] Orban M., Elsamanty M., Guo K., Zhang S., Yang H. (2022). A Review of Brain Activity and EEG-Based Brain–Computer Interfaces for Rehabilitation Application. Bioengineering.

[15] Park J., Park J., Shin D., Choi Y. (2021). A BCI Based Alerting System for Attention Recovery of UAV Operators. Sensors.

[16] Yang L., Van Hulle M.M. (2023). Real-Time Navigation in Google Street View^®^ Using a Motor Imagery-Based BCI. Sensors.

[17] Amprimo G., Rechichi I., Ferraris C., Olmo G. (2023). Measuring Brain Activation Patterns from Raw Single-Channel EEG during Exergaming: A Pilot Study. Electronics.

[18] Glavas K., Prapas G., Tzimourta K.D., Giannakeas N., Tsipouras M.G. (2022). Evaluation of the User Adaptation in a BCI Game Environment. Appl. Sci..

[19] Chang D., Xiang Y., Zhao J., Qian Y., Li F. (2022). Exploration of Brain-Computer Interaction for Supporting Children’s Attention Training: A Multimodal Design Based on Attention Network and Gamification Design. Int. J. Environ. Res. Public Health.

[20] Knierim M.T., Bleichner M.G., Reali P. (2023). A Systematic Comparison of High-End and Low-Cost EEG Amplifiers for Concealed, Around-the-Ear EEG Recordings. Sensors.

[21] Ferracuti F., Iarlori S., Mansour Z., Monteriù A., Porcaro C. (2022). Comparing between Different Sets of Preprocessing, Classifiers, and Channels Selection Techniques to Optimise Motor Imagery Pattern Classification System from EEG Pattern Recognition. Brain Sci..

[22] Baradaran F., Farzan A., Danishvar S., Sheykhivand S. (2023). Customized 2D CNN Model for the Automatic Emotion Recognition Based on EEG Signals. Electronics.

[23] Wang Y., Song C., Zhang T., Yao Z., Chang Z., Wang D. (2023). Feature Extraction of Motor Imagery EEG via Discrete Wavelet Transform and Generalized Maximum Fuzzy Membership Difference Entropy: A Comparative Study. Electronics.

[24] Ortega-Rodríguez J., Gómez-González J.F., Pereda E. (2023). Selection of the Minimum Number of EEG Sensors to Guarantee Biometric Identification of Individuals. Sensors.

[25] Cardona-Álvarez Y.N., Álvarez-Meza A.M., Cárdenas-Peña D.A., Castaño-Duque G.A., Castellanos-Dominguez G. (2023). A Novel OpenBCI Framework for EEG-Based Neurophysiological Experiments. Sensors.

[26] Saibene A., Caglioni M., Corchs S., Gasparini F. (2023). EEG-Based BCIs on Motor Imagery Paradigm Using Wearable Technologies: A Systematic Review. Sensors.

[27] Ali M.U., Kim K.S., Kallu K.D., Zafar A., Lee S.W. (2023). OptEF-BCI: An Optimization-Based Hybrid EEG and fNIRS–Brain Computer Interface. Bioengineering.

[28] Zafar A., Hussain S.J., Ali M.U., Lee S.W. (2023). Metaheuristic Optimization-Based Feature Selection for Imagery and Arithmetic Tasks: An fNIRS Study. Sensors.

[29] Erdoğan S.B., Yükselen G. (2022). Four-Class Classification of Neuropsychiatric Disorders by Use of Functional Near-Infrared Spectroscopy Derived Biomarkers. Sensors.

[30] Varandas R., Lima R., Bermúdez I Badia S., Silva H., Gamboa H. (2022). Automatic Cognitive Fatigue Detection Using Wearable fNIRS and Machine Learning. Sensors.

[31] Zapała D., Augustynowicz P., Tokovarov M. (2022). Recognition of Attentional States in VR Environment: An fNIRS Study. Sensors.

[32] Gulraiz A., Naseer N., Nazeer H., Khan M.J., Khan R.A., Shahbaz Khan U. (2022). LASSO Homotopy-Based Sparse Representation Classification for fNIRS-BCI. Sensors.

[33] Hamid H., Naseer N., Nazeer H., Khan M.J., Khan R.A., Shahbaz Khan U. (2022). Analyzing Classification Performance of fNIRS-BCI for Gait Rehabilitation Using Deep Neural Networks. Sensors.

[34] McClay W. (2018). A Magnetoencephalographic/Encephalographic (MEG/EEG) Brain-Computer Interface Driver for Interactive iOS Mobile Videogame Applications Utilizing the Hadoop Ecosystem, MongoDB, and Cassandra NoSQL Databases. Diseases.

[35] Reichert C., Dürschmid S., Kruse R., Hinrichs H. (2016). An Efficient Decoder for the Recognition of Event-Related Potentials in High-Density MEG Recordings. Computers.

[36] Dash D., Ferrari P., Dutta S., Wang J. (2020). NeuroVAD: Real-Time Voice Activity Detection from Non-Invasive Neuromagnetic Signals. Sensors.

[37] Xu F., Rong F., Miao Y., Sun Y., Dong G., Li H., Li J., Wang Y., Leng J. (2021). Representation Learning for Motor Imagery Recognition with Deep Neural Network. Electronics.

[38] Shokoueinejad M., Park D.-W., Jung Y.H., Brodnick S.K., Novello J., Dingle A., Swanson K.I., Baek D.-H., Suminski A.J., Lake W.B. (2019). Progress in the Field of Micro-Electrocorticography. Micromachines.

[39] Tasnim N., Ajam A., Ramos R., Koripalli M.K., Chennamsetti M., Choi Y. (2016). Handcrafted Electrocorticography Electrodes for a Rodent Behavioral Model. Technologies.

[40] Wolpaw J.R., McFarland D.J., Neat G.W., Forneris C.A. (1991). An EEG-based brain-computer interface for cursor control. Electroencephalogr. Clin. Neurophysiol..

[41] Nagel S., Spüler M. (2018). Modelling the brain response to arbitrary visual stimulation patterns for a flexible high-speed Brain-Computer Interface. PLoS ONE.

[42] Yuvaraj R., Thagavel P., Thomas J., Fogarty J., Ali F. (2023). Comprehensive Analysis of Feature Extraction Methods for Emotion Recognition from Multichannel EEG Recordings. Sensors.

[43] Damalerio R.B., Lim R., Gao Y., Zhang T.-T., Cheng M.-Y. (2023). Development of Low-Contact-Impedance Dry Electrodes for Electroencephalogram Signal Acquisition. Sensors.

[44] Shivaraja T.R., Remli R., Kamal N., Wan Zaidi W.A., Chellappan K. (2023). Assessment of a 16-Channel Ambulatory Dry Electrode EEG for Remote Monitoring. Sensors.

[45] Liu Q., Yang L., Zhang Z., Yang H., Zhang Y., Wu J. (2023). The Feature, Performance, and Prospect of Advanced Electrodes for Electroencephalogram. Biosensors.

[46] Liang H., Liu R. (2022). A New Generic Single-Channel Ear-EEG Recording Platform. Proceedings.

[47] Yuan H., Li Y., Yang J., Li H., Yang Q., Guo C., Zhu S., Shu X. (2021). State of the Art of Non-Invasive Electrode Materials for Brain–Computer Interface. Micromachines.

[48] Mwata-Velu T., Niyonsaba-Sebigunda E., Avina-Cervantes J.G., Ruiz-Pinales J., Velu-A-Gulenga N., Alonso-Ramírez A.A. (2023). Motor Imagery Multi-Tasks Classification for BCIs Using the NVIDIA Jetson TX2 Board and the EEGNet Network. Sensors.

[49] Al-Ayyad M., Owida H.A., De Fazio R., Al-Naami B., Visconti P. (2023). Electromyography Monitoring Systems in Rehabilitation: A Review of Clinical Applications, Wearable Devices and Signal Acquisition Methodologies. Electronics.

[50] Moontaha S., Schumann F.E.F., Arnrich B. (2023). Online Learning for Wearable EEG-Based Emotion Classification. Sensors.

[51] Mascia A., Collu R., Spanu A., Fraschini M., Barbaro M., Cosseddu P. (2023). Wearable System Based on Ultra-Thin Parylene C Tattoo Electrodes for EEG Recording. Sensors.

[52] Zhu H., Fu C., Shu F., Yu H., Chen C., Chen W. (2023). The Effect of Coupled Electroencephalography Signals in Electrooculography Signals on Sleep Staging Based on Deep Learning Methods. Bioengineering.

[53] Jakubowitz E., Feist T., Obermeier A., Gempfer C., Hurschler C., Windhagen H., Laves M.-H. (2023). Early Predictability of Grasping Movements by Neurofunctional Representations: A Feasibility Study. Appl. Sci..

[54] de Brito Guerra T.C., Nóbrega T., Morya E., de Martins A.M., de Sousa V.A. (2023). Electroencephalography Signal Analysis for Human Activities Classification: A Solution Based on Machine Learning and Motor Imagery. Sensors.

[55] Qiao Z., Van der Donck S., Moerkerke M., Dlhosova T., Vettori S., Dzhelyova M., van Winkel R., Alaerts K., Boets B. (2022). Frequency-Tagging EEG of Superimposed Social and Non-Social Visual Stimulation Streams Provides No Support for Social Salience Enhancement after Intranasal Oxytocin Administration. Brain Sci..

[56] Choi W., Kim M.-J., Yum M.-S., Jeong D.-H. (2022). Deep Convolutional Gated Recurrent Unit Combined with Attention Mechanism to Classify Pre-Ictal from Interictal EEG with Minimized Number of Channels. J. Pers. Med..

[57] Ehiabhi J., Wang H. (2023). A Systematic Review of Machine Learning Models in Mental Health Analysis Based on Multi-Channel Multi-Modal Biometric Signals. BioMedInformatics.

[58] Abdel-Hamid L. (2023). An Efficient Machine Learning-Based Emotional Valence Recognition Approach Towards Wearable EEG. Sensors.

[59] Doborjeh M., Liu X., Doborjeh Z., Shen Y., Searchfield G., Sanders P., Wang G.Y., Sumich A., Yan W.Q. (2023). Prediction of Tinnitus Treatment Outcomes Based on EEG Sensors and TFI Score Using Deep Learning. Sensors.

[60] Donisi L., Cesarelli G., Pisani N., Ponsiglione A.M., Ricciardi C., Capodaglio E. (2022). Wearable Sensors and Artificial Intelligence for Physical Ergonomics: A Systematic Review of Literature. Diagnostics.

[61] AL-Quraishi M.S., Elamvazuthi I., Tang T.B., Al-Qurishi M., Adil S.H., Ebrahim M. (2021). Bimodal Data Fusion of Simultaneous Measurements of EEG and fNIRS during Lower Limb Movements. Brain Sci..

[62] Rampp S., Kaltenhäuser M., Müller-Voggel N., Doerfler A., Kasper B.S., Hamer H.M., Brandner S., Buchfelder M. (2023). MEG Node Degree for Focus Localization: Comparison with Invasive EEG. Biomedicines.

[63] Fred A.L., Kumar S.N., Kumar Haridhas A., Ghosh S., Purushothaman Bhuvana H., Sim W.K.J., Vimalan V., Givo F.A.S., Jousmäki V., Padmanabhan P. (2022). A Brief Introduction to Magnetoencephalography (MEG) and Its Clinical Applications. Brain Sci..

[64] Morales Chacón L.M., González González J., Ríos Castillo M., Berrillo Batista S., Batista García-Ramo K., Santos Santos A., Quintanal Cordero N., Zaldívar Bermúdez M., Garbey Fernández R., Estupiñan Díaz B. (2021). Surgical Outcome in Extratemporal Epilepsies Based on Multimodal Pre-Surgical Evaluation and Sequential Intraoperative Electrocorticography. Behav. Sci..

[65] Seo J.-H., Tsuda I., Lee Y.J., Ikeda A., Matsuhashi M., Matsumoto R., Kikuchi T., Kang H. (2020). Pattern Recognition in Epileptic EEG Signals via Dynamic Mode Decomposition. Mathematics.

[66] Allison B.Z., Kübler A., Jin J. (2020). 30+ years of P300 brain–computer interfaces. Psychophysiology.

[67] Nakanishi M., Wang Y., Wang Y.T., Jung T.P. (2015). A comparison study of canonical correlation analysis based methods for detecting steady-state visual evoked potentials. PLoS ONE.

[68] Thomas E., Dyson M., Clerc M. (2013). An analysis of performance evaluation for motor-imagery based BCI. J. Neural Eng..

[69] Wang H., Yan F., Xu T., Yin H., Chen P., Yue H., Chen C., Zhang H., Xu L., He Y. (2021). Brain-controlled wheelchair review: From wet electrode to dry electrode, from single modal to hybrid modal, from synchronous to asynchronous. IEEE Access.

[70] Nooh A.A., Yunus J., Daud S.M. (2011). A review of asynchronous electroencephalogram-based brain computer interface systems. Int. Conf. Biomed. Eng. Technol. IPCBEE.

[71] Zhou Y., He S., Huang Q., Li Y. (2020). A hybrid asynchronous brain-computer interface combining SSVEP and EOG signals. IEEE Trans. Biomed. Eng..

[72] Delorme A., Sejnowski T., Makeig S. (2006). Enhanced detection of artifacts in EEG data using higher-order statistics and independent component analysis. NeuroImage.

[73] Mannan M.M.N., Kamran M.A., Jeong M.Y. (2018). Identification and Removal of Physiological Artifacts from Electroencephalogram Signals: A Review. IEEE Access.

[74] Urigüen J.A., Garcia-Zapirain B. (2015). EEG artifact removal—Stateof-the-art and guidelines. J. Neural Eng..

[75] Abdullah A.A., Zhang C.Z., Abdullah A., Lian S. (2014). Automatic Extraction System for Common Artifacts in EEG Signals Based on Evolutionary Stone’s BSS Algorithm. Math. Probl. Eng..

[76] Urigüen J.A., García-Zapirain B., Artieda J., Iriarte J., Valencia M. (2017). Comparison of background EEG activity of different groups of patients with idiopathic epilepsy using Shannon spectral entropy and cluster-based permutation statistical testing. PLoS ONE.

[77] Roy V., Shukla S., Shukla P.K., Rawat P. (2017). Gaussian Elimination-Based Novel Canonical Correlation Analysis Method for EEG Motion Artifact Removal. J. Healthc. Eng..

[78] Picton T.W., Van Roon P., Armilio M.L., Berg P., Ille N., Scherg M. (2000). The correction of ocular artifacts: A topographic perspective. Clin. Neurophysiol..

[79] Klados M.A., Papadelis C., Bamidis P.D., Braun C. (2011). REG-ICA: A hybrid methodology combining blind source separation and regression techniques for the rejection of ocular artifacts. Biomed. Signal Process. Control.

[80] Liu W., Park I., Wang Y., Principe J.C. (2009). Extended kernel recursive least squares algorithm. IEEE Trans. Signal Process..

[81] Mannan M.M., Jeong M.Y., Kamran M.A. (2016). Hybrid ICA-Regression: Automatic Identification and Removal of Ocular Artifacts from Electroencephalographic Signals. Front. Hum. Neurosci..

[82] Vapnik V.N. (1999). An overview of statistical learning theory. IEEE Transact. Neural Netw..

[83] Lotte F., Congedo M., Lecuyer A., Lamarche F., Arnaldi B. (2007). Review of classification algorithms for EEG based brain computer interfaces. J. Neural Eng..

[84] Rabiner L.R. (1989). A tutorial on hidden Markov models and selected applications in speech recognition. Proc. IEEE.

[85] Cerný V. (1985). Thermodynamical approach to the traveling salesman problem: An efficient simulation algorithm. J. Optim. Theory Appl..

[86] Obermeier B., Guger C., Neuper C., Pfurtscheller G. (2001). Hidden Markov models for online classification of single trial EEG data. Pattern Recognit. Lett..

[87] Cincotti F., Scipione A., Tiniperi A., Mattia D., Marciani A., Millan J., Salinari S., Bianchi L., Bablioni F. Comparison of different feature classifiers for brain computer interfaces. Proceedings of the First International IEEE EMBS Conference on Neural Engineering, 2003. Conference Proceedings.

[88] Pekša J. (2020). Autonomous Data-Driven Integration Algorithm. Proceedings of the 2020 4th International Conference on Cloud and Big Data Computing, ICCBDC ’20.

[89] Abiodun O.I., Jantan A., Omolara A.E., Dada K.V., Umar A.M., Linus O.U., Arshad H., Kazaure A.A., Gana U., Kiru M.U. (2019). Comprehensive review of artificial neural network applications to pattern recognition. IEEE Access.

[90] Zagirnyak M., Prus V. (2016). Use of neuronets in problems of forecasting the reliability of electric machines with a high degree of mean time between failures. Prz. Elektrotechniczny (Electr. Rev.).

[91] Ko W., Jeon E., Jeong S., Suk H.I. (2021). Multi-scale neural network for EEG representation learning in BCI. IEEE Comput. Intell. Mag..

[92] LeCun Y., Boser B., Denker J.S., Henderson D., Howard R.E., Hubbard W., Jackel L.D. (1989). Backpropagation Applied to Handwritten Zip Code Recognition. Neural Comput..

[93] Schirrmeister R.T., Springenberg J.T., Fiederer L.D.J., Glasstetter M., Eggensperger K., Tangermann M., Hutter F., Burgard W., Ball T. (2017). Deep learning with convolutional neural networks for EEG decoding and visualization. Hum. Brain Mapp..

[94] Christou V., Miltiadous A., Tsoulos I., Karvounis E., Tzimourta K.D., Tsipouras M.G., Anastasopoulos N., Tzallas A.T., Giannakeas N. (2022). Evaluating the Window Size’s Role in Automatic EEG Epilepsy Detection. Sensors.

[95] Cerasa A., Tartarisco G., Bruschetta R., Ciancarelli I., Morone G., Calabrò R.S., Pioggia G., Tonin P., Iosa M. (2022). Predicting Outcome in Patients with Brain Injury: Differences between Machine Learning versus Conventional Statistics. Biomedicines.

[96] Naebi A., Feng Z., Hosseinpour F., Abdollahi G. (2021). Dimension Reduction Using New Bond Graph Algorithm and Deep Learning Pooling on EEG Signals for BCI. Appl. Sci..

[97] Łysiak A., Paszkiel S. (2021). A Method to Obtain Parameters of One-Column Jansen–Rit Model Using Genetic Algorithm and Spectral Characteristics. Appl. Sci..

[98] Z-Flores E., Trujillo L., Legrand P., Faïta-Aïnseba F. (2020). EEG Feature Extraction Using Genetic Programming for the Classification of Mental States. Algorithms.

[99] Hag A., Handayani D., Altalhi M., Pillai T., Mantoro T., Kit M.H., Al-Shargie F. (2021). Enhancing EEG-Based Mental Stress State Recognition Using an Improved Hybrid Feature Selection Algorithm. Sensors.

[100] Gao Y., Si J., Wu S., Li W., Liu H., Chen J., He Q., Zhang Y. (2021). Improvement of the Classification Accuracy of Steady-State Visual Evoked Potential-Based Brain-Computer Interfaces by Combining L1-MCCA with SVM. Appl. Sci..

[101] Li Z., Qiu L., Li R., He Z., Xiao J., Liang Y., Wang F., Pan J. (2020). Enhancing BCI-Based Emotion Recognition Using an Improved Particle Swarm Optimization for Feature Selection. Sensors.

[102] Majidov I., Whangbo T. (2019). Efficient Classification of Motor Imagery Electroencephalography Signals Using Deep Learning Methods. Sensors.

[103] Reñosa C.R.M., Bandala A.A., Vicerra R.R.P. Classification of Confusion Level Using EEG Data and Artificial Neural Networks. Proceedings of the 2019 IEEE 11th International Conference on Humanoid, Nanotechnology, Information Technology, Communication and Control, Environment, and Management (HNICEM).

[104] Sareen E., Singh L., Varkey B., Achary K., Gupta E. (2020). EEG dataset of individuals with intellectual and developmental disorder and healthy controls under rest and music stimuli. Data Brief.

[105] Malete T.N., Moruti K., Thapelo T.S., Jamisola R.S. EEG-based Control of a 3D Game Using 14-channel Emotiv Epoc^+^. Proceedings of the 2019 IEEE International Conference on Cybernetics and Intelligent Systems (CIS) and IEEE Conference on Robotics, Automation and Mechatronics (RAM).

[106] Peterson V., Galván C., Hernández H., Saavedra M.P., Spies R. (2022). A motor imagery vs. rest dataset with low-cost consumer grade EEG hardware. Data Brief.

[107] Lee H.K., Choi Y.-S. A convolution neural networks scheme for classification of motor imagery EEG based on wavelet time-frequecy image. Proceedings of the 2018 International Conference on Information Networking (ICOIN).

[108] Goldberger A.L., Amaral L.A., Glass L., Hausdorff J.M., Ivanov P.C., Mark R.G., Mietus J.E., Moody G.B., Peng C.-K., Stanley H.E. (2000). PhysioNet: Components of a new research resource for complex physiologic signals. Circulation.

[109] Blankertz B., Muller K.-R., Krusienski D., Schalk G., Wolpaw J., Schlogl A., Pfurtscheller G., Millan J., Schroder M., Birbaumer N. (2006). The BCI competition III: Validating alternative approaches to actual BCI problems. IEEE Trans. Neural Syst. Rehabil. Eng..

[110] Tangermann M., Müller K.-R., Aertsen A., Birbaumer N., Braun C., Brunner C., Leeb R., Mehring C., Miller K.J., Müller-Putz G.R. (2012). Review of the BCI competition IV. Front. Neurosci..

[111] Hajipour Sardouie S., Shamsollahi M.B. (2012). Selection of efficient features for discrimination of hand movements from MEG using a BCI competition IV data set. Front. Neurosci..

[112] Stober S., Avital S., Owen A.M., Grahn J.A. (2015). Towards Music Imagery Information Retrieval: Introducing the OpenMIIR Dataset of EEG Recordings from Music Perception and Imagination. ISMIR.

[113] Altuwaijri G.A., Muhammad G., Altaheri H., Alsulaiman M. (2022). A Multi-Branch Convolutional Neural Network with Squeeze-and-Excitation Attention Blocks for EEG-Based Motor Imagery Signals Classification. Diagnostics.

[114] Altuwaijri G.A., Muhammad G. (2022). A Multibranch of Convolutional Neural Network Models for Electroencephalogram-Based Motor Imagery Classification. Biosensors.

[115] Hafeez T., Umar Saeed S.M., Arsalan A., Anwar S.M., Ashraf M.U., Alsubhi K. (2021). EEG in game user analysis: A framework for expertise classification during gameplay. PLoS ONE.

[116] Bano K.S., Bhuyan P., Ray A. EEG-Based Brain Computer Interface for Emotion Recognition. Proceedings of the 2022 5th International Conference on Computational Intelligence and Networks (CINE).

[117] Luján M.Á., Jimeno M.V., Mateo Sotos J., Ricarte J.J., Borja A.L. (2021). A Survey on EEG Signal Processing Techniques and Machine Learning: Applications to the Neurofeedback of Autobiographical Memory Deficits in Schizophrenia. Electronics.

[118] Niedermeyer E., da Silva F.L. (2005). Electroencephalography: Basic Principles, Clinical Applications, and Related Fields.

[119] Cajigas I., Davis K.C., Meschede-Krasa B., Prins N.W., Gallo S., Naeem J.A., Palermo A., Wilson A., Guerra S., Parks B.A. (2021). Implantable brain–computer interface for neuroprosthetic-enabled volitional hand grasp restoration in spinal cord injury. Brain Commun..

[120] Lim J., Lin D., Sohn W.J., McCrimmon C.M., Wang P.T., Nenadic Z., Do A.H. (2022). BCI-Based Neuroprostheses and Physiotherapies for Stroke Motor Rehabilitation. Reinkensmeyer, Neurorehabilitation Technology.

[121] Sanna A., Manuri F., Fiorenza J., De Pace F. (2022). BARI: An Affordable Brain-Augmented Reality Interface to Support Human–Robot Collaboration in Assembly Tasks. Information.

[122] Shieh C.-P., Yang S.-H., Liu Y.-S., Kuo Y.-T., Lo Y.-C., Kuo C.-H., Chen Y.-Y. (2020). Simultaneously Spatiospectral Pattern Learning and Contaminated Trial Pruning for Electroencephalography-Based Brain Computer Interface. Symmetry.

[123] Xu B., Li W., He X., Wei Z., Zhang D., Wu C., Song A. (2020). Motor Imagery Based Continuous Teleoperation Robot Control with Tactile Feedback. Electronics.

[124] Tayeb Z., Fedjaev J., Ghaboosi N., Richter C., Everding L., Qu X., Wu Y., Cheng G., Conradt J. (2019). Validating Deep Neural Networks for Online Decoding of Motor Imagery Movements from EEG Signals. Sensors.

[125] Edelman B.J., Meng J., Suma D., Zurn C., Nagarajan E., Baxter B.S., Cline C.C., He B.J.S.R. (2019). Noninvasive neuroimaging enhances continuous neural tracking for robotic device control. Sci. Robot..

[126] Wu S.-J., Nicolaou N., Bogdan M. (2020). Consciousness Detection in a Complete Locked-in Syndrome Patient through Multiscale Approach Analysis. Entropy.

[127] Powers J.C., Bieliaieva K., Wu S., Nam C.S. (2015). The Human Factors and Ergonomics of P300-Based Brain-Computer Interfaces. Brain Sci..

[128] Xu B., Li W., Liu D., Zhang K., Miao M., Xu G., Song A. (2022). Continuous Hybrid BCI Control for Robotic Arm Using Noninvasive Electroencephalogram, Computer Vision, and Eye Tracking. Mathematics.

[129] Dumitrescu C., Costea I.-M., Semenescu A. (2021). Using Brain-Computer Interface to Control a Virtual Drone Using Non-Invasive Motor Imagery and Machine Learning. Appl. Sci..

[130] Shah U., Alzubaidi M., Mohsen F., Abd-Alrazaq A., Alam T., Househ M. (2022). The Role of Artificial Intelligence in Decoding Speech from EEG Signals: A Scoping Review. Sensors.

[131] Ron-Angevin R., Fernández-Rodríguez Á., Dupont C., Maigrot J., Meunier J., Tavard H., Lespinet-Najib V., André J.-M. (2023). Comparison of Two Paradigms Based on Stimulation with Images in a Spelling Brain–Computer Interface. Sensors.

[132] Akram F., Alwakeel A., Alwakeel M., Hijji M., Masud U. (2022). A Symbols Based BCI Paradigm for Intelligent Home Control Using P300 Event-Related Potentials. Sensors.

[133] Velasco-Álvarez F., Fernández-Rodríguez Á., Vizcaíno-Martín F.-J., Díaz-Estrella A., Ron-Angevin R. (2021). Brain–Computer Interface (BCI) Control of a Virtual Assistant in a Smartphone to Manage Messaging Applications. Sensors.

[134] Mannan M.M.N., Kamran M.A., Kang S., Choi H.S., Jeong M.Y. (2020). A Hybrid Speller Design Using Eye Tracking and SSVEP Brain–Computer Interface. Sensors.

[135] Anumanchipalli G.K., Chartier J., Chang E.F. (2019). Speech synthesis from neural decoding of spoken sentences. Nature.

[136] Willett F.R., Avansino D.T., Hochberg L.R., Henderson J.M., Shenoy K.V. (2021). High-performance brain-to-text communication via handwriting. Nature.

[137] Cabañero-Gómez L., Hervas R., Bravo J., Rodriguez-Benitez L. (2018). Computational EEG Analysis Techniques When Playing Video Games: A Systematic Review. Proceedings.

[138] Choi H., Lim H., Kim J.W., Kang Y.J., Ku J. (2019). Brain Computer Interface-Based Action Observation Game Enhances Mu Suppression in Patients with Stroke. Electronics.

[139] Paszkiel S., Rojek R., Lei N., Castro M.A. (2021). A Pilot Study of Game Design in the Unity Environment as an Example of the Use of Neurogaming on the Basis of Brain–Computer Interface Technology to Improve Concentration. NeuroSci.

[140] Cattan G., Mendoza C., Andreev A., Congedo M. (2018). Recommendations for Integrating a P300-Based Brain Computer Interface in Virtual Reality Environments for Gaming. Computers.

[141] Ahn M., Lee M., Choi J., Jun S.C. (2014). A Review of Brain-Computer Interface Games and an Opinion Survey from Researchers, Developers and Users. Sensors.

[142] Sung Y., Cho K., Um K. (2012). A Development Architecture for Serious Games Using BCI (Brain Computer Interface) Sensors. Sensors.

[143] Kovyazina M.S., Varako N.A., Lyukmanov R.K., Asiatskaya G.A., Suponeva N.A., Trofimova A.K. (2019). Neurofeedback in the Rehabilitation of Patients with Motor Disorders after Stroke. Hum. Physiol..

[144] TajDini M., Sokolov V., Kuzminykh I., Shiaeles S., Ghita B. (2020). Wireless Sensors for Brain Activity—A Survey. Electronics.

[145] Serrano-Barroso A., Siugzdaite R., Guerrero-Cubero J., Molina-Cantero A.J., Gomez-Gonzalez I.M., Lopez J.C., Vargas J.P. (2021). Detecting Attention Levels in ADHD Children with a Video Game and the Measurement of Brain Activity with a Single-Channel BCI Headset. Sensors.

[146] Bulat M., Karpman A., Samokhina A., Panov A. (2020). Playing a P300-based BCI VR game leads to changes in cognitive functions of healthy adults. bioRxiv.

[147] Kohli V., Tripathi U., Chamola V., Rout B.K., Kanhere S.S. (2022). A review on Virtual Reality and Augmented Reality use-cases of Brain Computer Interface based applications for smart cities. Microprocess. Microsyst..

[148] Al-Nafjan A., Aldayel M. (2022). Predict Students’ Attention in Online Learning Using EEG Data. Sustainability.

[149] Rácz M., Noboa E., Détár B., Nemes Á., Galambos P., Szűcs L., Márton G., Eigner G., Haidegger T. (2022). PlatypOUs—A Mobile Robot Platform and Demonstration Tool Supporting STEM Education. Sensors.

[150] Balderas D., Ponce P., Lopez-Bernal D., Molina A. (2021). Education 4.0: Teaching the Basis of Motor Imagery Classification Algorithms for Brain-Computer Interfaces. Future Internet.

[151] Burgos D. (2020). Motor Imagery Experiment Using BCI: An Educational Technology Approach. Radical Solutions and Learning Analytics.

[152] Teo S.H., Poh X.W., Lee T.S., Guan C., Cheung Y.B., Fung D.S., Zhang H.H., Chin Z.Y., Wang C.C., Sung M. (2021). Brain-computer interface based attention and social cognition training programme for children with ASD and co-occurring ADHD: A feasibility trial. Res. Autism Spectr. Disord..

[153] Hadjiaros M., Neokleous K., Shimi A., Avraamides M.N., Pattichis C.S. (2023). Virtual Reality Cognitive Gaming Based on Brain Computer Interfacing: A Narrative Review. IEEE Access.

[154] Ramírez-Moreno M.A., Carrillo-Tijerina P., Candela-Leal M.O., Alanis-Espinosa M., Tudón-Martínez J.C., Roman-Flores A., Ramírez-Mendoza R.A., Lozoya-Santos J.D.J. (2021). Evaluation of a Fast Test Based on Biometric Signals to Assess Mental Fatigue at the Workplace—A Pilot Study. Int. J. Environ. Res. Public Health.

[155] Lim C.G., Soh C.P., Lim S.S.Y., Fung D.S.S., Guan C., Lee T.-S. (2023). Home-based brain–computer interface attention training program for attention deficit hyperactivity disorder: A feasibility trial. Child Adolesc. Psychiatry Ment. Health.

[156] Jia Z., Cai X., Jiao Z. (2022). Multi-Modal Physiological Signals Based Squeeze-and-Excitation Network with Domain Adversarial Learning for Sleep Staging. IEEE Sens. J..

[157] Chen T., Huang H., Pan J., Li Y. An EEG-based brain-computer interface for automatic sleep stage classification. Proceedings of the 13th IEEE Conference on Industrial Electronics and Applications (ICIEA).

[158] Abenna S., Nahid M., Bouyghf H. (2022). Sleep Stages Detection Based BCI: A Novel Single-Channel EEG Classification Based on Optimized Bandpass Filter. Advanced Technologies for Humanity. ICATH 2021. Lecture Notes on Data Engineering and Communications Technologies.

[159] Jia Z., Lin Y., Wang J., Ning X., He Y., Zhou R., Zhou Y., Li-wei H.L. (2021). Multi-view spatial-temporal graph convolutional networks with domain generalization for sleep stage classification. IEEE Trans. Neural Syst. Rehabil. Eng..

[160] Eldele E., Chen Z., Liu C., Wu M., Kwoh C.K., Li X., Guan C. (2021). An attention-based deep learning approach for sleep stage classification with single-channel EEG. IEEE Trans. Neural Syst. Rehabil. Eng..

[161] Michielli N., Acharya U.R., Molinari F. (2019). Cascaded LSTM recurrent neural network for automated sleep stage classification using single-channel EEG signals. Comput. Biol. Med..

[162] Santaji S., Desai V. (2020). Analysis of EEG signal to classify sleep stages using machine learning. Sleep Vigil.

